# Master of Science in Medical Statistics Programme at Universiti Sains Malaysia: 20 Years Ongoing

**DOI:** 10.21315/mjms2023.30.1.1

**Published:** 2023-02-28

**Authors:** Bachok Norsa’adah, Najib Majdi Yaacob, Sarimah Abdullah, Cheng Kueh Yee, Siti Azrin Ab Hamid, Anis Kausar Ghazali, Wan Nor Arifin

**Affiliations:** Unit of Biostatistics and Research Methodology, School of Medical Sciences, Universiti Sains Malaysia, Kelantan, Malaysia

**Keywords:** Medical Statistics, Biostatistics, Master’s degree, Universiti Sains Malaysia

## Abstract

Health and medical research are important parts of the curriculum of medical and health programmes in universities and play an important role in the functioning of organisations related to health care. There is a shortage of well-trained health and medical research statisticians. This article describes the courses and structure of the Master of Science in Medical Statistics programme at Universiti Sains Malaysia (USM), as well as the graduates’ achievements. It is a 2-year programme that prepares qualified and competent graduates in statistical methods and data analysis for research in health and medical sciences. The Biostatistics and Research Methodology Unit, School of Medical Sciences, USM has been running the programme since 2003. It is currently the only medical statistics programme available in Malaysia. There have been 97 graduates since 2005, with an employment rate of 96.7% and a successful subsequent doctorate rate of 21.1%. Most of the students returned to their previous employments, mainly with the Ministry of Health of Malaysia and several others became lecturers, statisticians or research officers. The employability of graduates from this programme is very high and their professional future is bright. We hope our graduates will impart their knowledge and skills to the nation.

## Introduction

The Master of Science (MSc) in Medical Statistics programme aims to impart in-depth knowledge and skills in the application of statistical methods and the analysis of research data related to health and medicine (1). This programme is designed to produce a competent graduate in medical statistics to work effectively as an important collaborator in investigating health-related issues. Successful graduates are expected to have careers as medical statisticians in academia, research organisations and the pharmaceutical industry. This programme also offers the opportunity to pursue studies in the field of medical statistics at the postgraduate level.

Most university programmes related to health and medical sciences, including undergraduate, master and doctorate degrees, have made it mandatory to conduct research and submit a thesis. Medical statistics is a crucial tool in health and medical research, patient management decision-making and public health administration. Generally, knowledge of medical statistics among students and staff is inadequate (2). There is a strong demand for consultants in statistical and research methods, especially in Malaysia. Consultation can prevent misuse of statistical methods, mismatch between research objectives and study design, and misinterpretation of statistical software output. Quality publication in high impact journals also requires sophisticated statistical methods and data analysis.

This article reviews the MSc in Medical Statistics programme at Universiti Sains Malaysia (USM) from its first enrolment until the current enrolment. We describe the programme courses and structure, and the graduate accomplishments.

### Programme Structure

The programme is a mixed mode that spans four semesters over a two-year period, with coursework in the first year, and research and a thesis in the second year. The first two semesters have 10 credit units each and the last two semesters have 20 credit units, for a total of 40 units for the whole programme (1). The first semester consists of three courses: i) Basic Statistics; ii) Intermediate Statistics and iii) Principles of Epidemiology, while the second semester consists of four courses: i) Critical Appraisal; ii) Clinical Epidemiology; iii) Research Methodology and Protocol Development and iv) Advanced Statistics. The third and fourth semesters are meant for conducting research and writing the thesis. This programme provides knowledge of epidemiology, research methods and medical statistics. This knowledge will be used to implement research and write a thesis, as well as to provide client consultation in the field of medical and health research.

### Assessments

There are two forms of assessment: continuous assessment and final examination (1). Continuous assessment contributes 30% to the overall marks, comprising assignments, presentations and tasks in the form of software and/or quiz. Meanwhile, 70% of the total mark comes from the final examination held at the end of each semester. As of 2017, the passing grade for a course is C+ or 2.33 Grade Point Average (GPA), while the minimum Cumulative Grade Point Average (CGPA) for graduation is 3.00. Students who fail any course in the first two semesters or do not achieve at least 3.00 CGPA may retake the courses offered during the long semester break (*Kursus Semasa Cuti Panjang, KSCP*) or in the following semesters.

### Teaching and Learning Methods

Before the COVID-19 pandemic, students were expected to attend face-to-face classes. The movement control order was introduced on 18 March 2020 and lifted in stages until 1 April 2022. For the remainder of 2020, throughout 2021, and the first semester of 2022, all teaching and learning was delivered through online and e-learning methods. The shift to online teaching and learning methods was a challenging time for all lecturers. Many adaptations and innovations in the use of technology in teaching had to be implemented quickly to ensure programme continuity during the pandemic. From the second semester in October 2022, blended teaching methods are being practiced, meaning online teaching is mixed with conventional face-to-face teaching. The programme could be improved by learning from other universities that offer continuous online education for applied statistics (3).

In accordance with the directive by the Ministry of Higher Education, MQA Bil 7/2022 dated 8 August 2022, teaching and learning should be maximised by blended or hybrid methods starting from semester 2 of the academic year 2022/2023. For the MSc in Medical Statistics programme, some topics that do not require hands-on learning have been delivered online synchronously or asynchronously through an e-learning website. Topics requiring hands-on and interactive engagement with lecturers were conducted face-to-face. In the future, the teaching methods for medical statistics should be flexible and diverse (2), including problem-based learning and continuing education trainings.

### Research Project

In the second semester, students present their research projects to a panel of lecturers. Students have three research options: i) primary data research; ii) secondary data research and iii) systematic review and meta-analysis. Each student is given a supervisor, who helps the student choose a research topic suitable to be carried out within the given time frame. Primary data research means that students collect the data and perform the statistical analysis to answer the proposed research questions. Secondary data research emphasises on data analysis and discussion of the results, utilising data collected by other researchers for different purposes. Students are also allowed to conduct a systematic review and meta-analysis.

### Student Activities

Students are expected to participate in hands-on practical sessions on relevant topics, particularly sessions on statistical analyses. They must be able to apply their knowledge, determine the appropriate statistical analysis for a given dataset, and interpret and present the results. Students must master the use of statistical software, especially SPSS, R, STATA and Mplus. Students are trained to critically appraise research proposals and scientific papers. Students are also encouraged to present their research findings at a conference before graduating and write at least one manuscript before the *viva voce* session.

**Figure 1:**
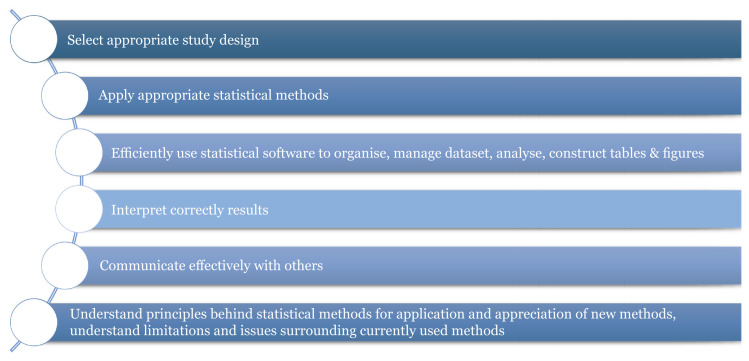
Roles of medical statistician

Since there is a high demand for statistical consultation on campus, students are encouraged to do consultation for research projects, especially advising on sample size determination and statistical analyses. After the first year, students are effectively in an internship period, during which they develop skills in advising researchers in statistics and research methodology, as well as developing their communication skills by providing these services, while under supervision. Statistical consultation is intended to help students develop statistical thinking and intuition and train them in statistical practice. Figure 1 shows several roles expected of a medical statistician. The study design must be consistent with the research objectives. Statisticians are expected to apply appropriate statistical methods, use statistical software efficiently and interpret results correctly. Communication between the statistician and the clients is extremely important. Statisticians will recommend solutions based on their understanding of the issues from the information provided by the client. On the other hand, the clients must have a sufficient understanding of the recommendations to be able to solve the problems.

### Programme Staff

The programme is led by a programme coordinator, assisted by the first- and second-year coordinators and all lecturers. This is a team of seven active lecturers responsible for teaching and learning of the programme. It consists of one professor, two associate professors and four senior lecturers. Five of the lecturers have doctoral degrees and two have master’s degrees. Five lecturers are also practising medical doctors. All lecturers are trained in medical statistics and four have additional expertise in epidemiology.

### Background of Students

**Figure 2:**
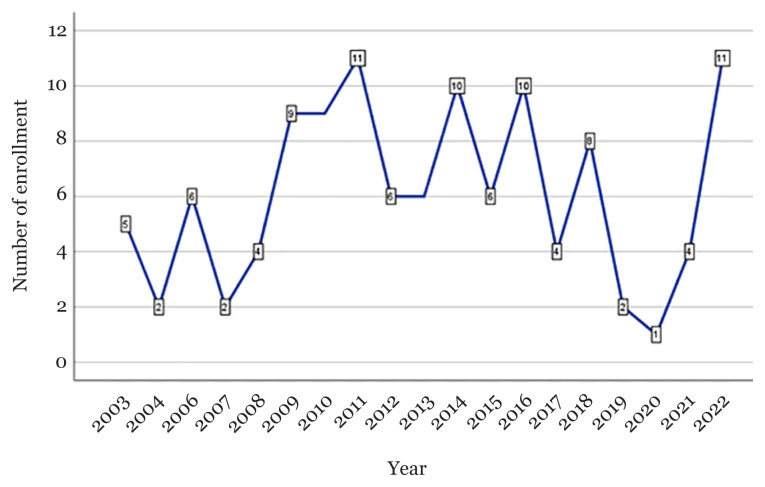
Number of MSc Medical Statistics students enrolled in 2003–2022

The MSc in Medical Statistics programme started in 2003. Figure 2 shows that 116 students have enrolled in the programme, with only in 2005 having no student. The maximum enrolment was 11 students in 2011 and 2022. Regarding the students’ bachelor’s degrees, 34 (29.3%) had a bachelor’s degree in Statistics, 23 (19.8%) in Pharmacy, 12 (10.3%) in Nursing, 10 (8.6%) in Biomedicine, 10 (8.6%) in Medicine and 7 (6.0%) in Mathematics. Fifty-four students (46.6%) received sponsorship from the governments. Most of the students were Malaysians, with 86 (74.1%) were Malays and 19 (16.4%) were Chinese. There were four international students: two Nigerians, one Uzbek and one Palestinian. Most students (93 [80.2%]) were females. Forty-nine students (42.2%) had prior employment and 61 students (52.6%) were fresh graduates, with 50 (43.1%) students aged less than 25 years old. The oldest student was 48 years old. The mean (SD) age was 27.7 (5.3) years old.

### Graduate Performance

**Figure 3:**
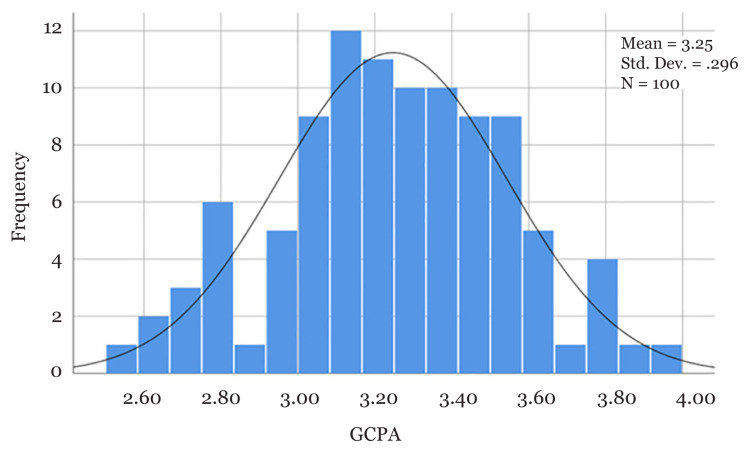
Distribution of GCPA score by MSc Medical Statistics students

**Figure 4:**
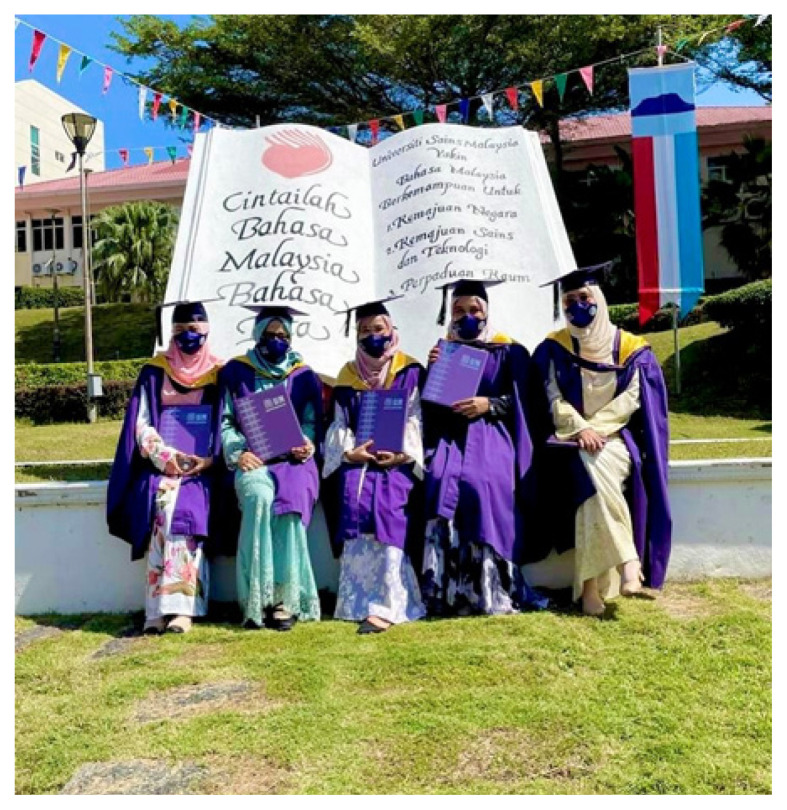
Students of Batch 2018 during convocation

**Figure 5:**
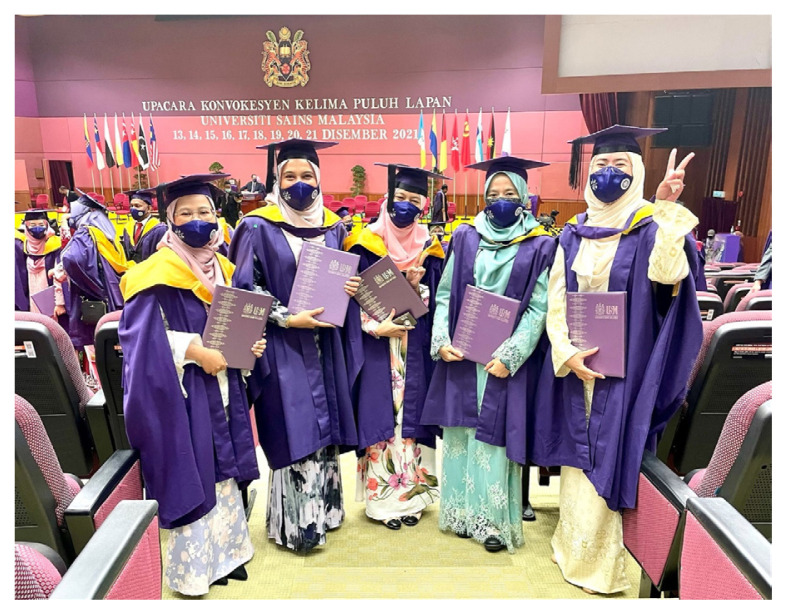
Students of Batch 2018 during convocation

**Figure 6:**
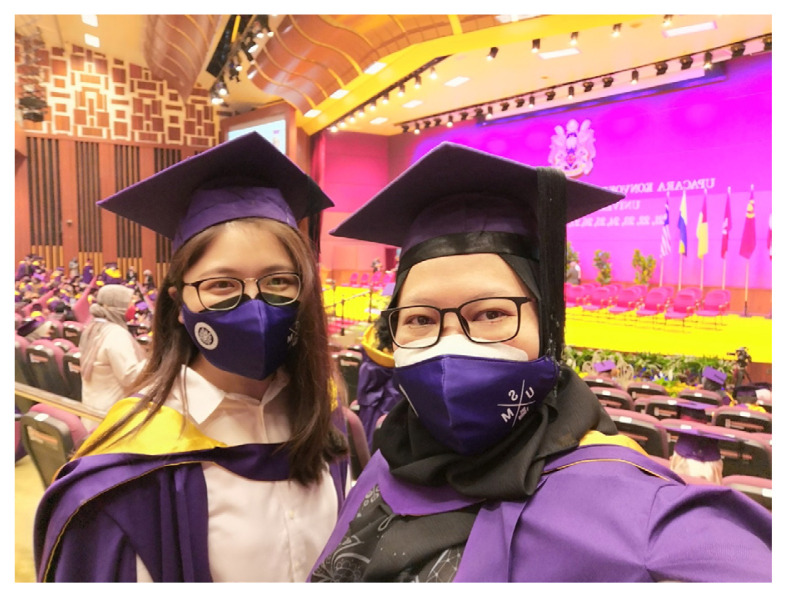
Students of Batch 2019 during convocation

**Figure 7:**
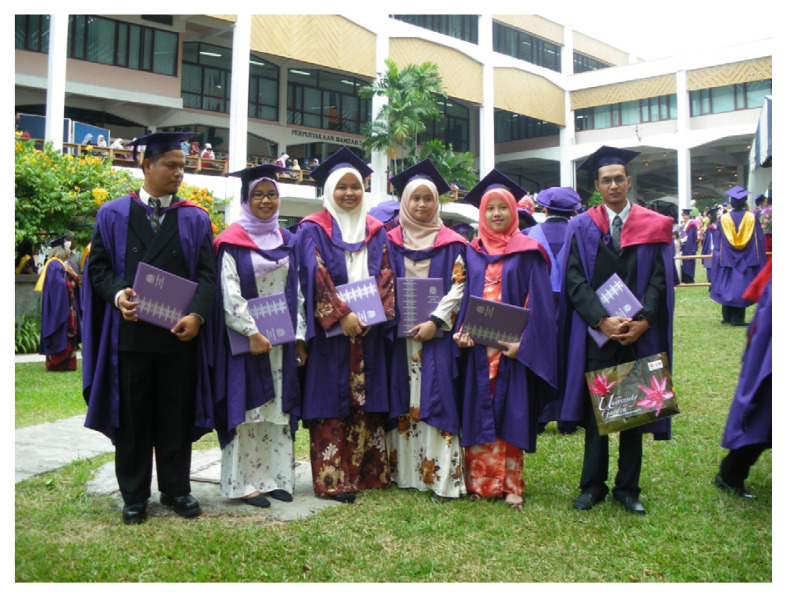
Students of Batch 2006 during convocation

**Figure 8:**
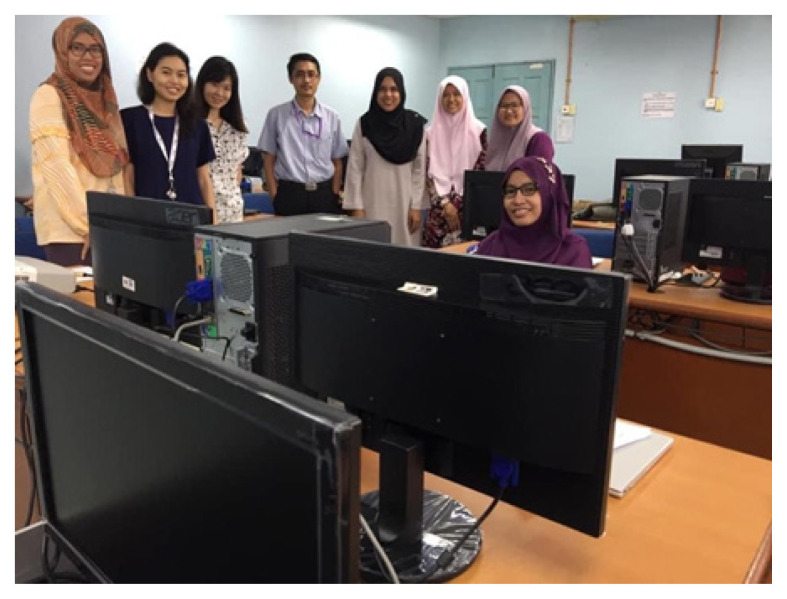
Students in computer statistical lab

**Figure 9:**
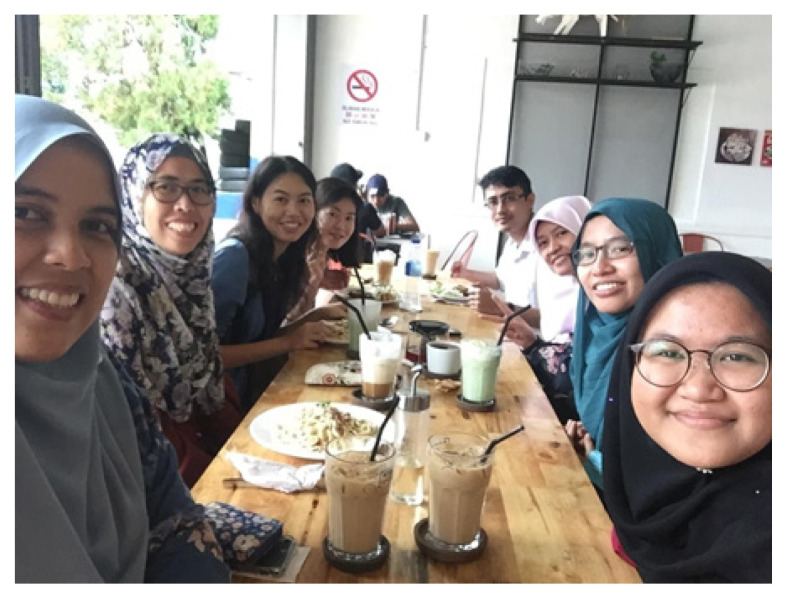
Students bonding during lunch time

The first graduation took place in 2005. In 17 years, there were 97 successful graduates and four students who did not graduate. One student was terminated due to poor cumulative grades and three withdrew from the programme. Among graduates, only five students (5.15%) did not graduate on time (GOT). Figure 3 shows that the mean (SD) of CGPA score was 3.24 (0.29). The highest CGPA score was 3.93 and 15 (15.5%) of students scored more than 3.50. Figures 4, 5, 6 and 7 are graduate students at convocations. Figure 8 is a picture of students in the statistical computing lab after a teaching and learning session. Figure 9 shows the students were having lunch.

### Graduate Employment

Out of 97 graduates, we were unable to contact seven of them. Of the 90 graduates, 87 (96.7%) were employed, 19 (21.1%) successfully completed subsequent doctoral degrees and an additional 13 (14.4%) were studying for a PhD. Most students returned to their previous employment, mainly with the Ministry of Health Malaysia, having studied medical statistics to improve their work efficiency. Twenty-three of the graduates became lecturers at public universities and nine at private universities. Twenty-four were statisticians or research officers at the Ministry of Health Malaysia, the Department of Statistics, universities and private companies. Of those who have worked, only 8 (9.2%) worked in an unrelated field.

## Conclusion

The MSc in Medical Statistics programme is relevant to the current academic situation in Malaysia. The programme provides competent, skilled medical statisticians who serve the nation. Graduates are mainly employed in medical and health research at various governmental and private institutions. The employability of graduates from this programme is very high, with a bright future career.
